# Supplementary figures and images for: Two-step mechanism of J-domain action in driving Hsp70 function
Source: PLoS Comput Biol. 2020 Jun 1;16(6):e1007913. doi: 10.1371/journal.pcbi.1007913 (PMC7289447; doi:10.1371/journal.pcbi.1007913)

**S1 Fig**

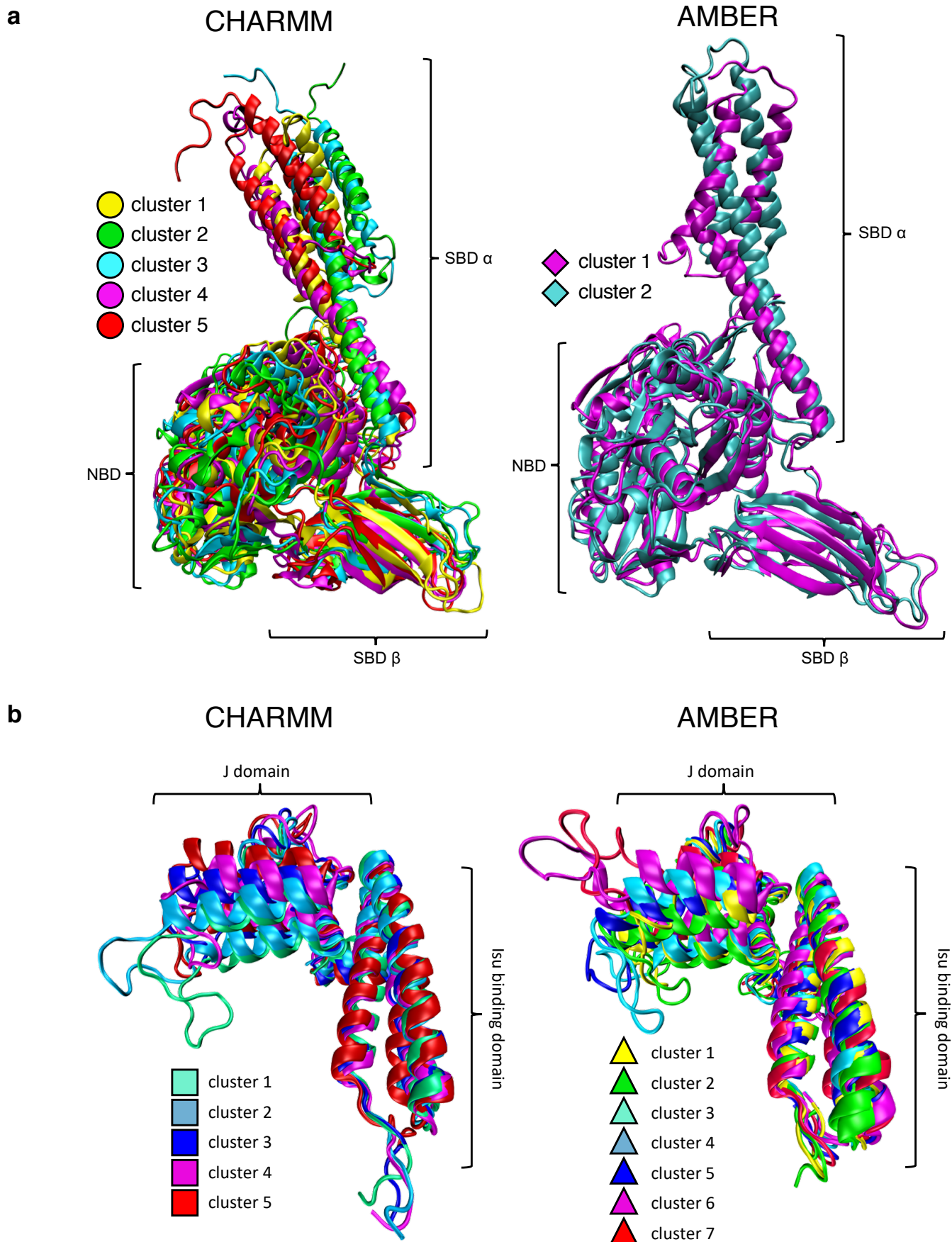

Supplement: S1 Fig — Representative structures of Ssq1 (a) and Hsc20 (b) from our unbiased molecular dynamics simulations carried out using the CHARMM36 (left) and AMBER99SB-ILDN (right) force fields. Ssq1 and Hsc20 were simulated for 2 and 10 μs, respectively, with CHARMM, and for 2 and 5 μs, respectively, with AMBER. The obtained trajectories were subject to cluster analysis with an RMSD cutoff of 0.7 nm (Ssq1) and 0.35 nm (Hsc20). The centroids of each cluster were superimposed and are shown in different colors. NBD, SBDa and SBDb domains (in Ssq1) and J-domain and Isu1 binding domain (in Hsc20) are indicated. (PDF) [file pcbi.1007913.s001.pdf]

S2 Fig

a

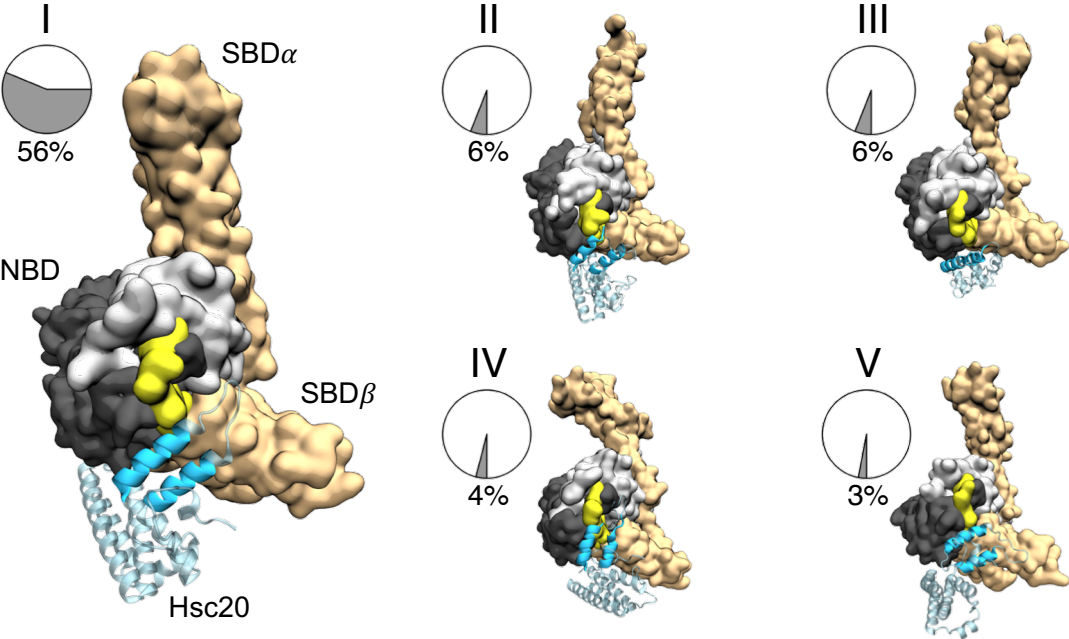

b

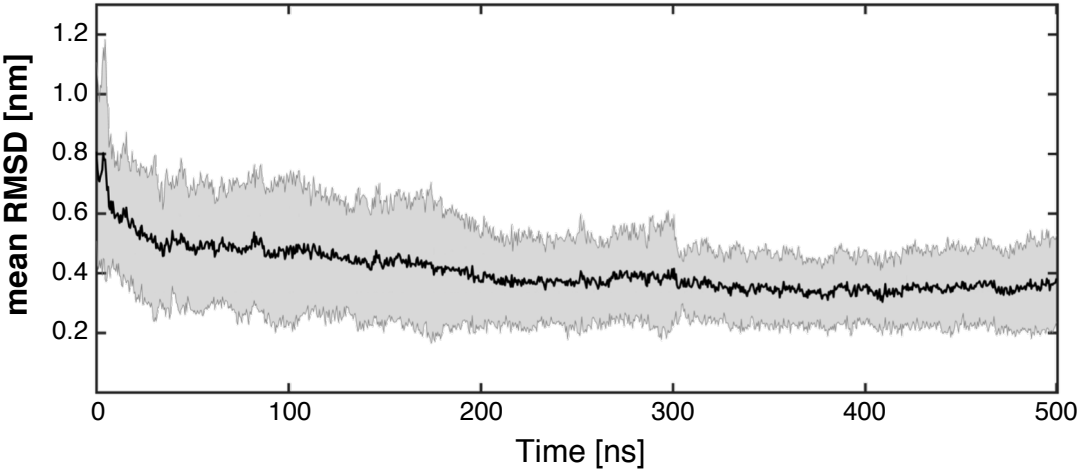

Supplement: S2 Fig — (a) Five most populated binding poses of Hsc20 along with their percentage contributions to the bound- state ensemble of the Hsc20-Ssq1 complex, obtained by the combined docking/MD simulation approach. Helices II and III are shown in cyan and the rest of Hsc20 in transparent cyan. SBD is in light brown, Ia and IIa subdomains of NBD are in light and dark grey, respectively, linker in yellow. (b) Time evolution of the average RMSD of helices II and III of the J-domain with respect to the center of the most populated binding pose above (the dominant bound state). Averaging was done over all 18 independent MD trajectories that were found to visit the dominant bound state at least once over the course of 500 ns. The shaded area shows the standard deviation of the RMSD. (PDF) [file pcbi.1007913.s002.pdf]

S3 Fig

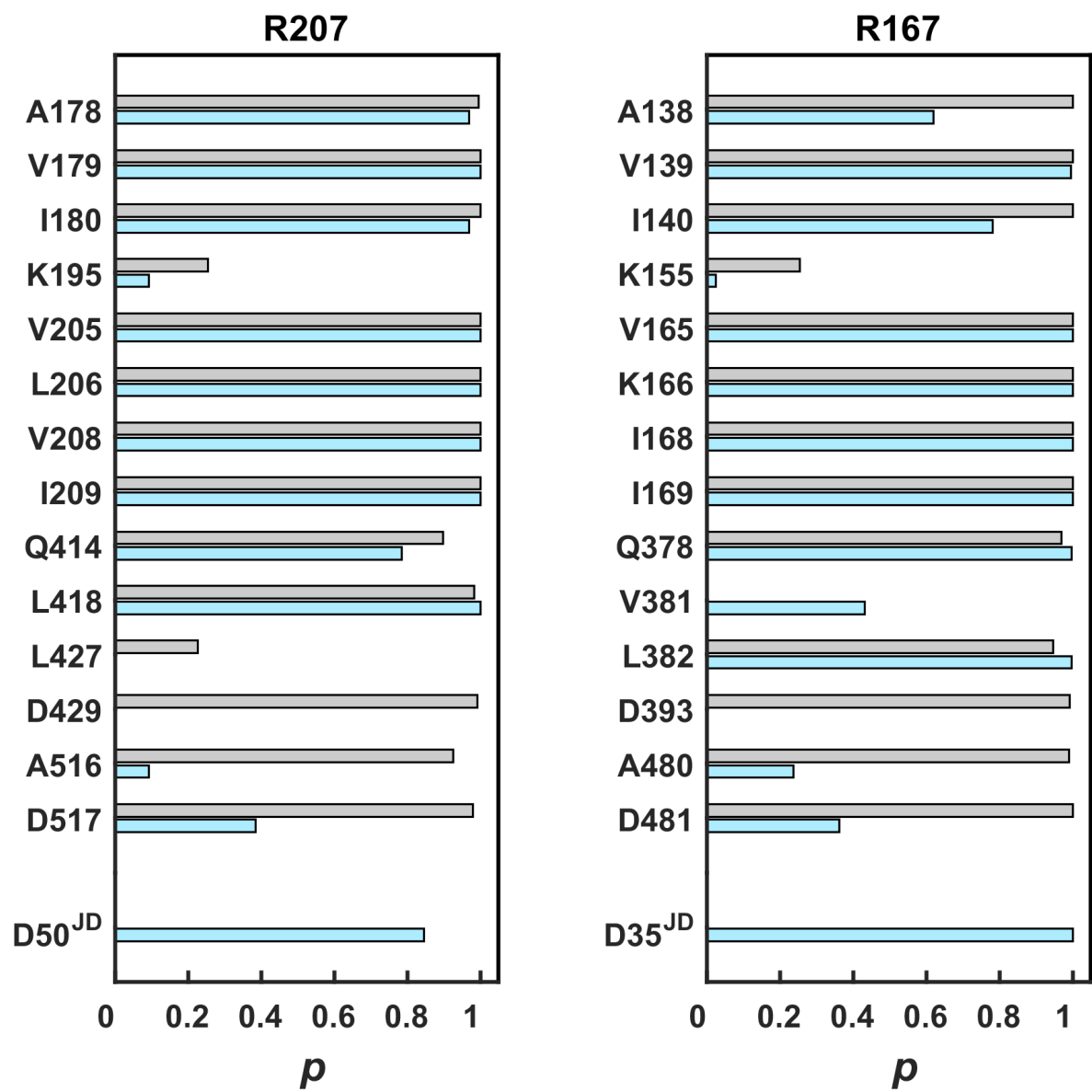

Supplement: S3 Fig — Contact maps of Ssq1 R207 and DnaK R167 calculated from 2D metadynamics trajectories in absence (grey bars) and presence (cyan bars) of Hsc20 and DnaJ J-domain, respectively. The contact is defined with a cutoff distance of 0.5 nm between non-hydrogen atoms, and the contacts with probability above 20% in either state are shown. (PDF) [file pcbi.1007913.s003.pdf]

**S4 Fig**

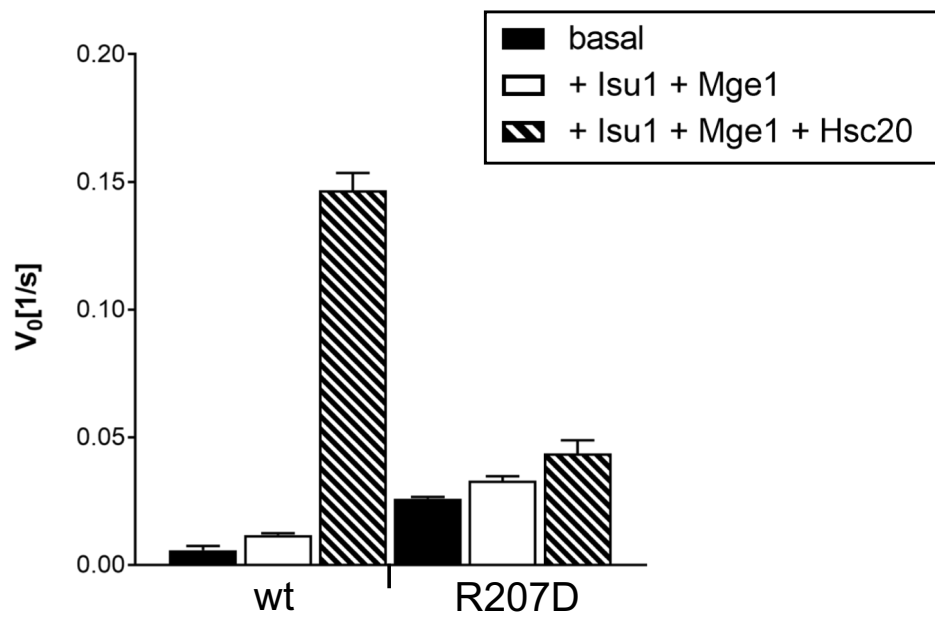

Supplement: S4 Fig — Steady-state ATPase activity of Ssq1 was measured alone, or in the presence of indicated proteins, using an enzymatically coupled assay. Bars represent average value for three independent measurements with error bars as standard deviation. Concentration of components: 1 μM Ssq1; 3 μM Hsc20; 3 μM Isu1; 1 μM Mge1; 1 mM ATP. (PDF) [file pcbi.1007913.s004.pdf]

**S5 Fig**

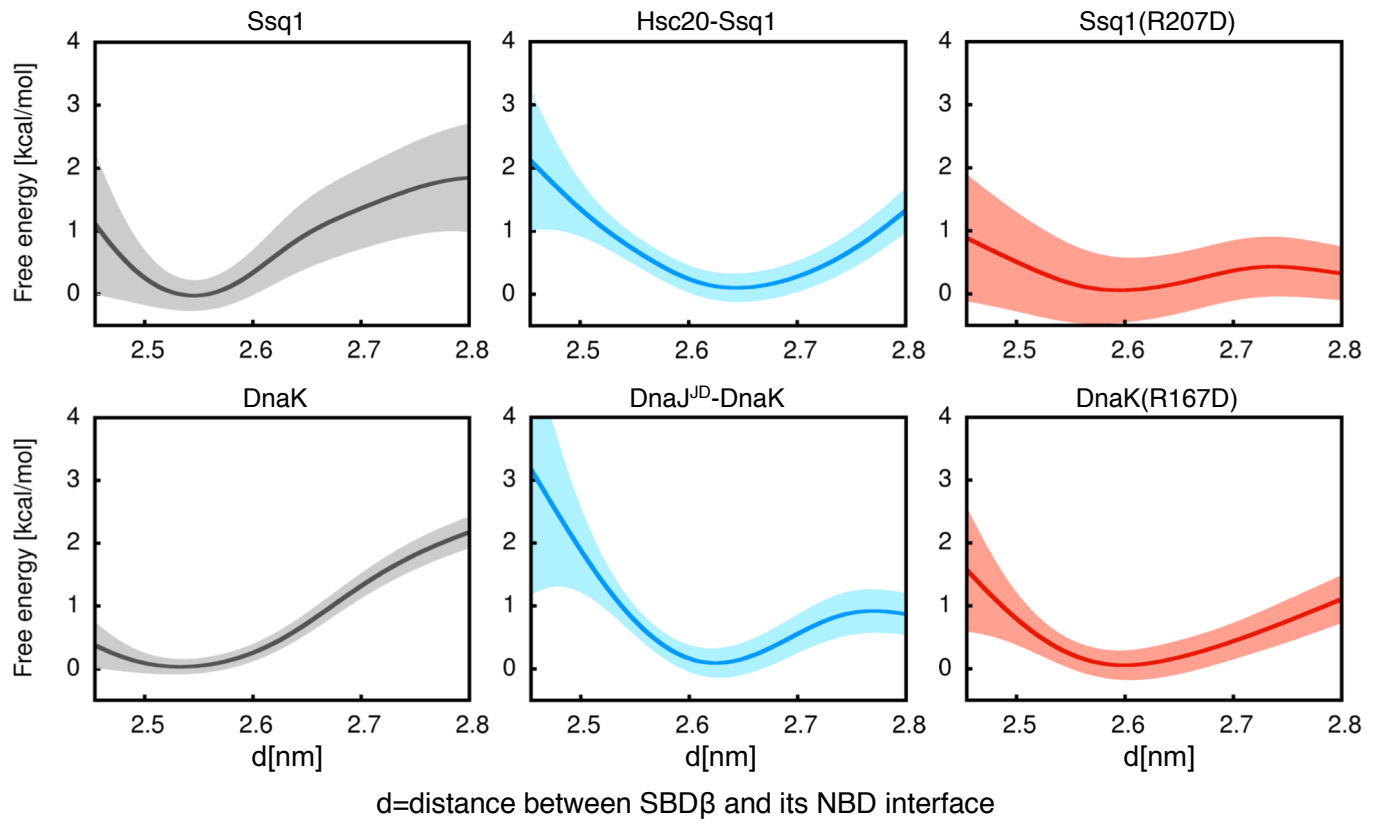

Supplement: S5 Fig — (PDF) [file pcbi.1007913.s005.pdf]

S6 Fig

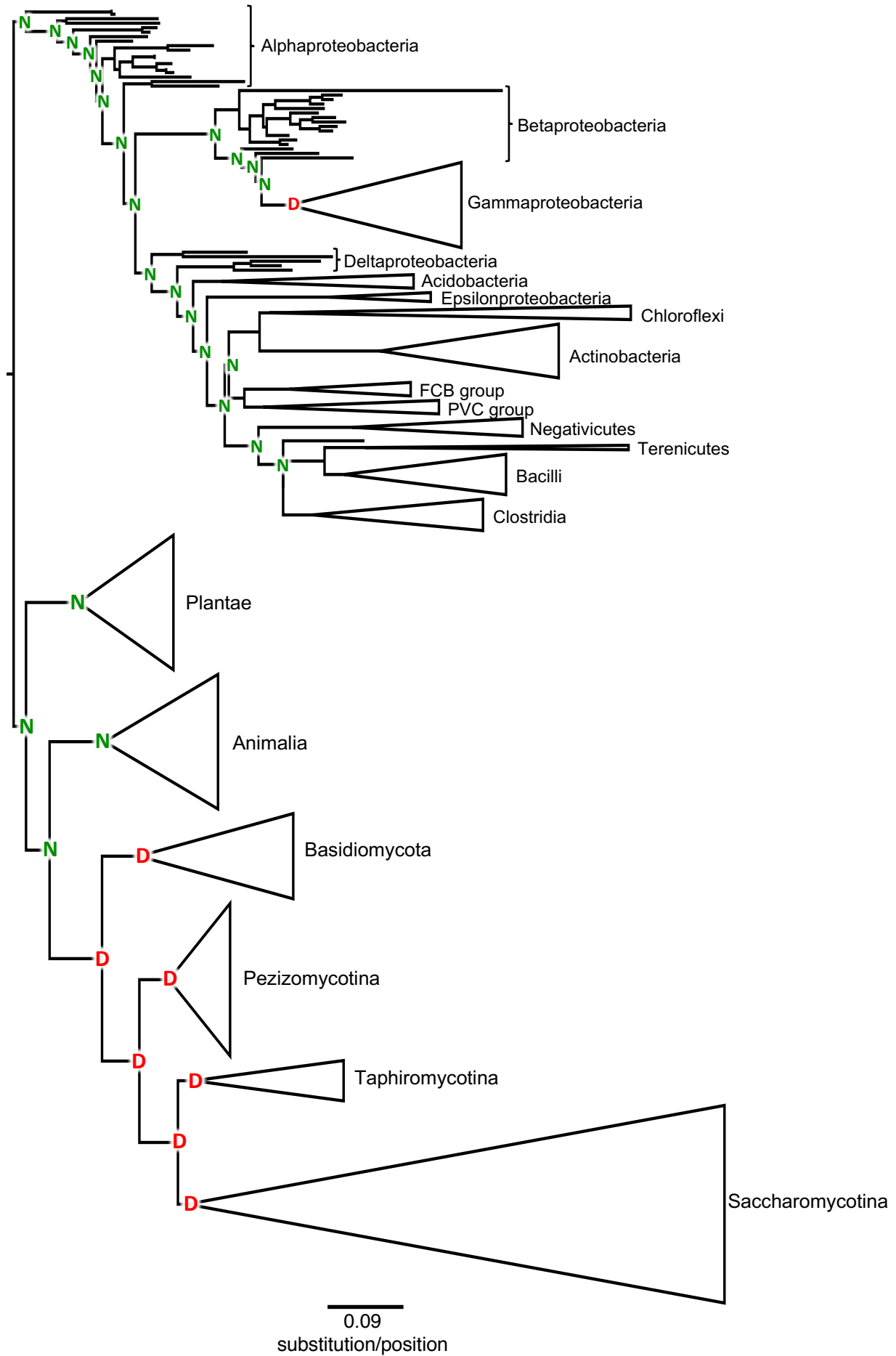

Supplement: S6 Fig — Scale is in amino acid substitutions per position. (PDF) [file pcbi.1007913.s006.pdf]

S8 Fig

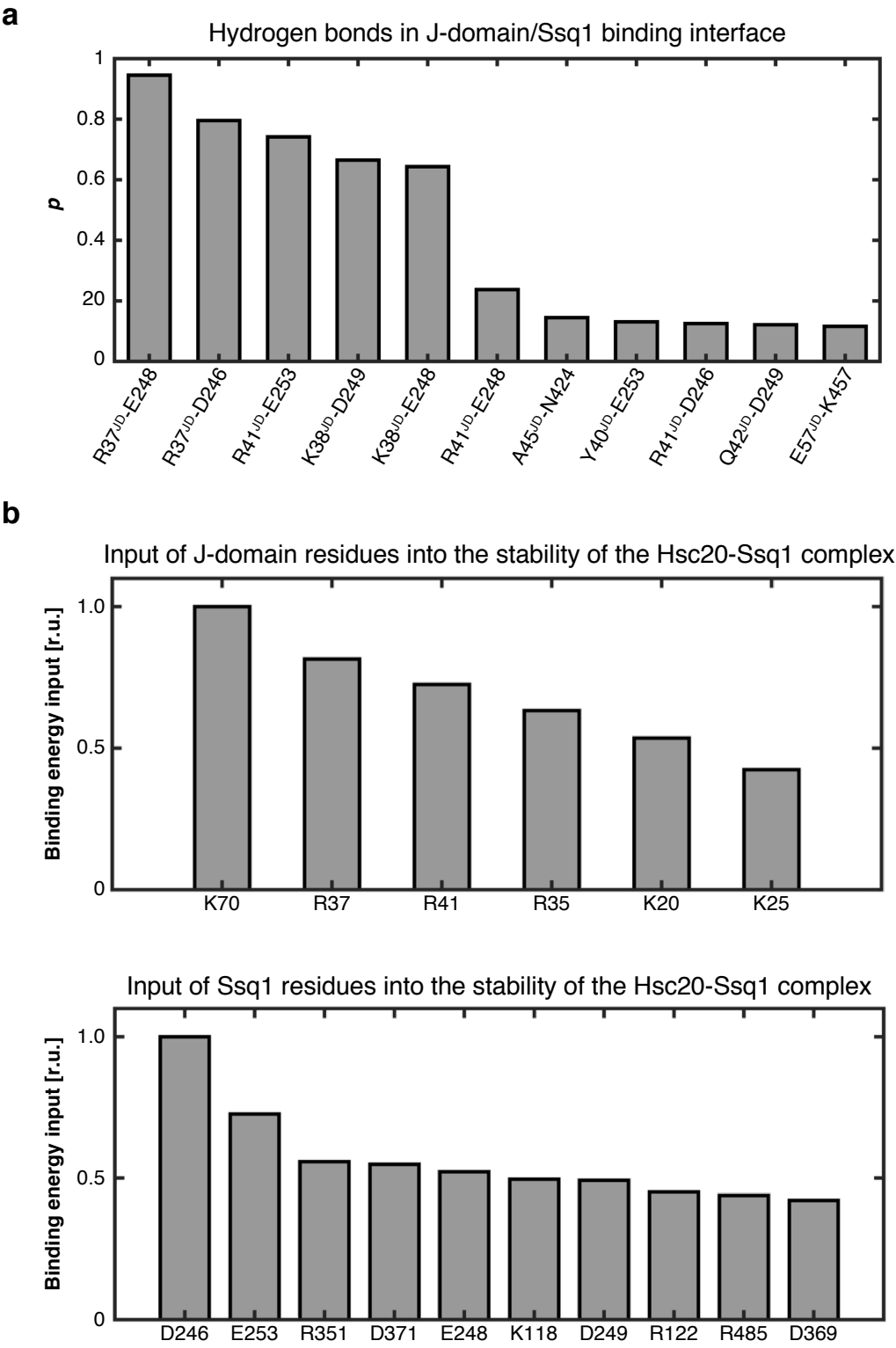

Supplement: S8 Fig — (a) Probabilities (p) of the most stable hydrogen bonds across the J-domain/Ssq1 interface, calculated from the 10.5 μs trajectory of the dominant bound state. (b) Relative energy inputs to Hsc20-Ssq1 complex binding energy calculated with MM/PBSA for the residues of the Hsc20 J-domain (top) and Ssq1 (bottom). (PDF) [file pcbi.1007913.s008.pdf]

S9 Fig

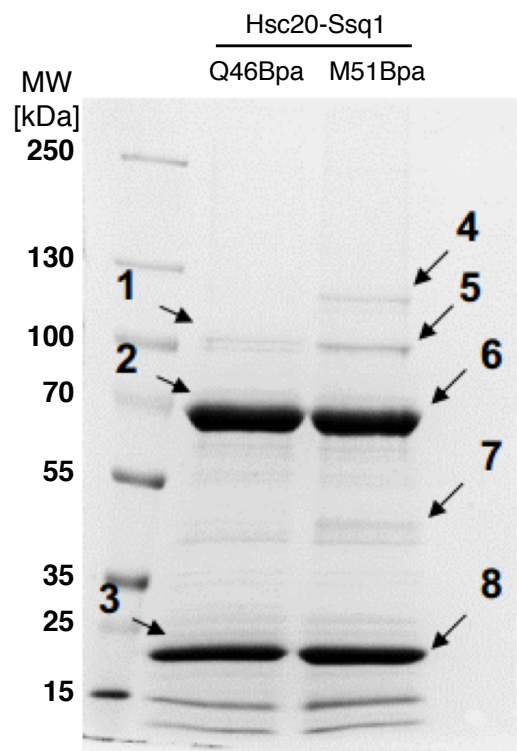

| Band | Protein | MW [Da] | MASCOT Score | emPAI |
|------|---------|---------|--------------|-------|
| 1    | Ssq1    | 72378   | 8231         | 14.51 |
|      | Hsc20   | 21822   | 1658         | 7.14  |
| 2    | Ssq1    | 72378   | 38546        | 40.80 |
| 3    | Hsc20   | 21822   | 10706        | 10.92 |
|      | Ssq1    | 72378   | 10259        | 11.28 |
| 4    | Hsc20   | 21822   | 13640        | 16.43 |
|      | Ssq1    | 72378   | 2637         | 7.14  |
| 5    | Ssq1    | 72378   | 13350        | 18.48 |
|      | Hsc20   | 21822   | 2790         | 7.14  |
| 6    | Ssq1    | 72378   | 34076        | 26.79 |
| 7    | Ssq1    | 72378   | 14297        | 15.44 |
|      | Hsc20   | 21822   | 3283         | 7.14  |
| 8    | Hsc20   | 21822   | 9675         | 10.92 |
|      | Ssq1    | 72738   | 3798         | 7.16  |

Supplement: S9 Fig — (left) Purified Hsc20 Q46Bpa and M51Bpa variants, were incubated with Ssq1 WT in the presence of ATP, irradiated with UV light and separated by SDS-PAGE. Indicated bands (1–8) were excised and subjected to trypsin digestion followed by LC-MS. Migrations of size standard, in kDa, are indicated. (right) Identification of proteins detected in individual SDS-PAGE bands by Mascot, along with statistical significance of Peptide Mass Fingerprint search (Mascot score) and protein abundance estimation (emPAI). (PDF) [file pcbi.1007913.s009.pdf]

S10 Fig

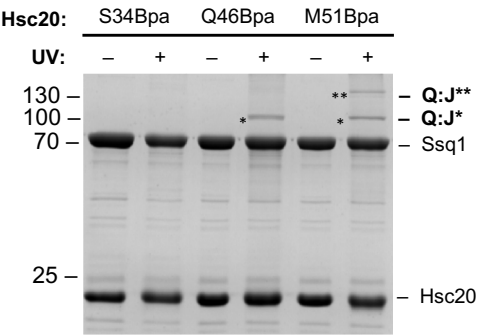

Supplement: S10 Fig — Purified Hsc20 S34Bpa, Q46Bpa and M51Bpa variants, as indicated, were incubated with purified Ssq1 in the presence of ATP. After UV irradiation (+), or as a control no irradiation (-), reaction mixtures were separated by SDS-PAGE. Migrations of size standard, in kDa, are indicated; Q:J* and Q:J** indicate positions of the Ssq1-Hsc20 crosslinks, which were identified using mass-spectroscopy (S9 Fig). (PDF) [file pcbi.1007913.s010.pdf]

**S11 Fig**

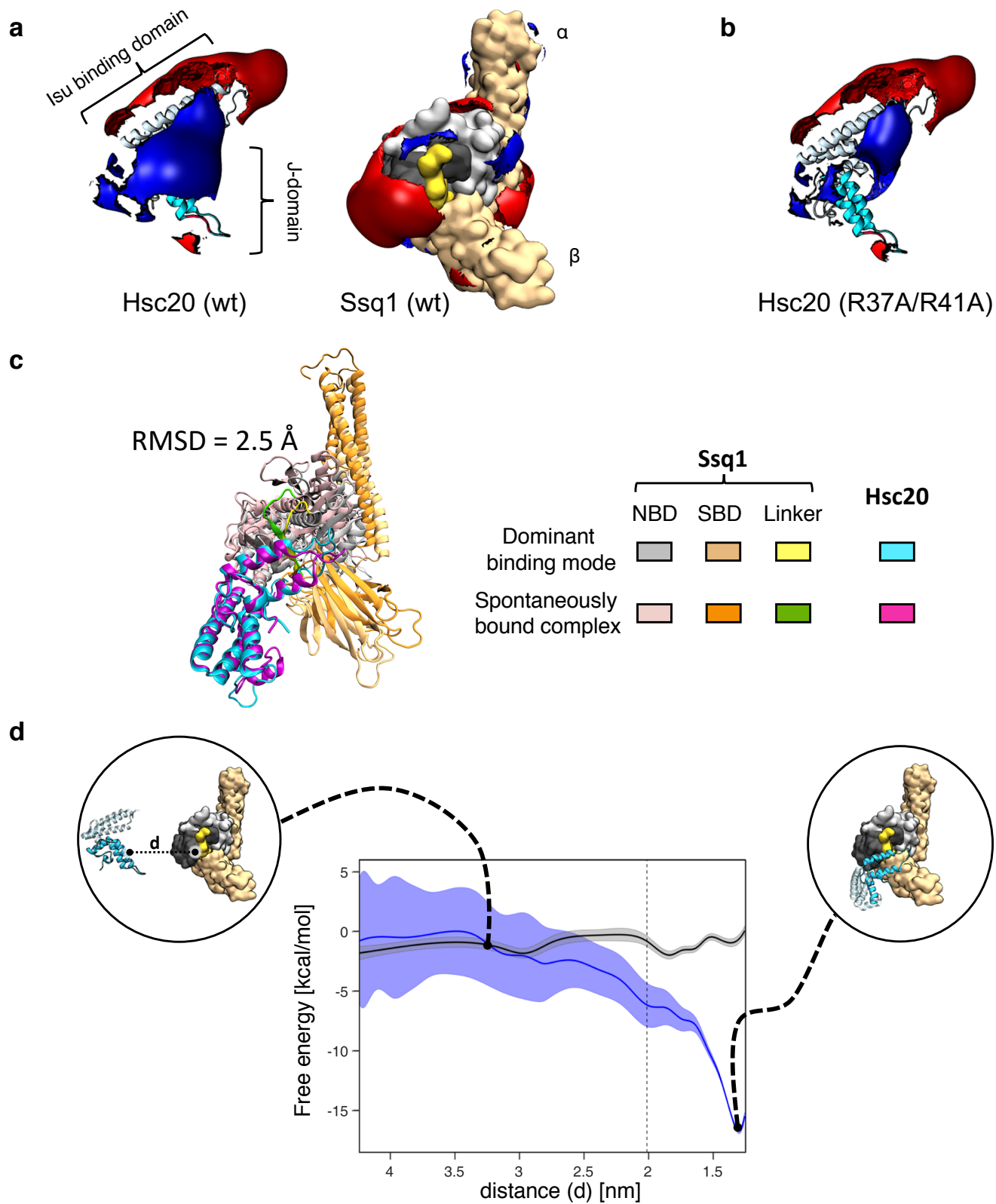

Supplement: S11 Fig — Hsc20/Ssq1 recognition is driven by long-range electrostatic forces (a) Electrostatic isopotential contours at +/- 1kT/e (blue and red, respectively) around Hsc20 and Ssq1 and (b) around Hsc20 double substitution variant R37A,R41A. Coloring of structures as in Fig 1: Hsc20: J-domain (cyan); Isu binding domain (light blue); Ssq1: SBDα and SBDβ (brown); interdomain linker (yellow); NBD subdomains Ia and IIa in light and dark grey, respectively. (c) Structural alignment of Hsc20-Ssq1 complex obtained by spontaneous binding MD simulations and the dominant bound state obtained by molecular docking/MD simulations. The heavy-atom RMSD for J-domains after aligning NBDs equals to 2.5 Å. (d) Free energy profiles for Hsc20-Ssq1 binding as a function of separation distance, d, between J-domain of Hsc20 and NBD subdomain IIa of Ssq1. Binding of Hsc20 wild-type (WT) (blue line), binding of Hsc20(R37A,R41A) variant (red line); shaded areas show the standard error. The vertical dashed line, demarcating the bund and unbound states, indicates the distance beyond which all specific interactions between the binding partners are lost. Representative structures are shown in circles: unbound state (left), bound state—corresponding to the energy well (right). (PDF) [file pcbi.1007913.s011.pdf]

S12 Fig

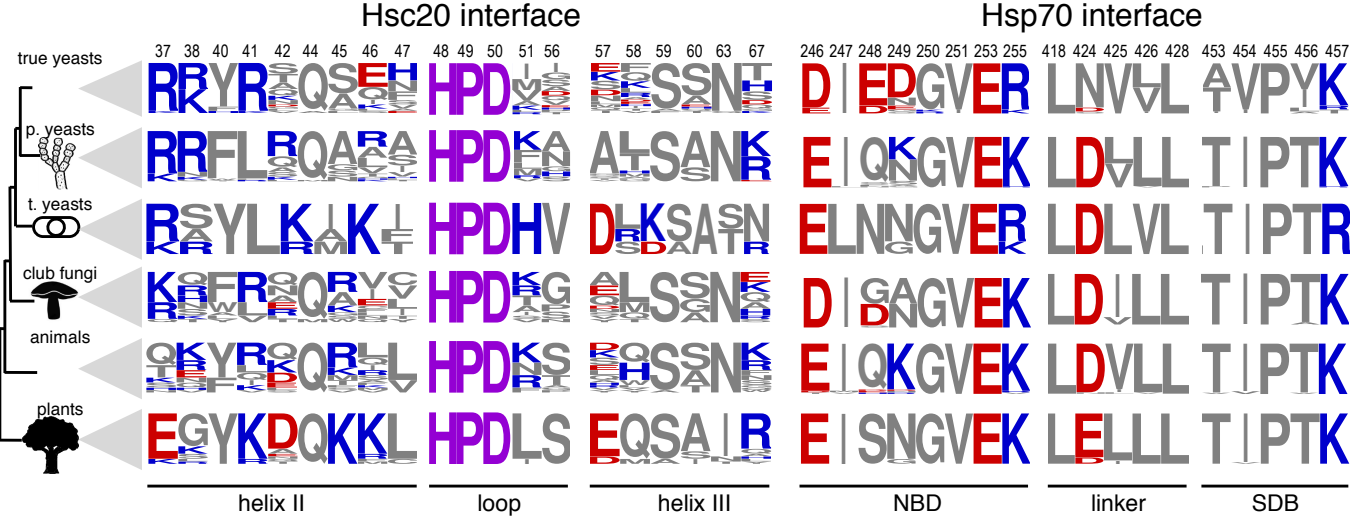

Supplement: S12 Fig — Sequence variability of positions that form the Hsc20/Ssq1 binding interfaces (positions shown in green in Fig 5A) for orthologs of Hsc20 (left) and orthologs of Ssq1 (right) from the following taxonomic groups: Saccharomycotina (true yeasts), Pezizomycotina (p. yeasts), Taphiromycotina (t. yeasts), Basidiomycota (club fungi), Animals (animals) and Plants (plants). Sequence logos represent the amino acid frequency of each interfacial position in the orthologs examined, with positively charged (blue), negatively charged (red) and uncharged (grey) residues. Position numbering is that for S. cerevisiae. Phylogenetic relationships among taxonomic groups are depicted on the left. (PDF) [file pcbi.1007913.s012.pdf]

S13 Fig

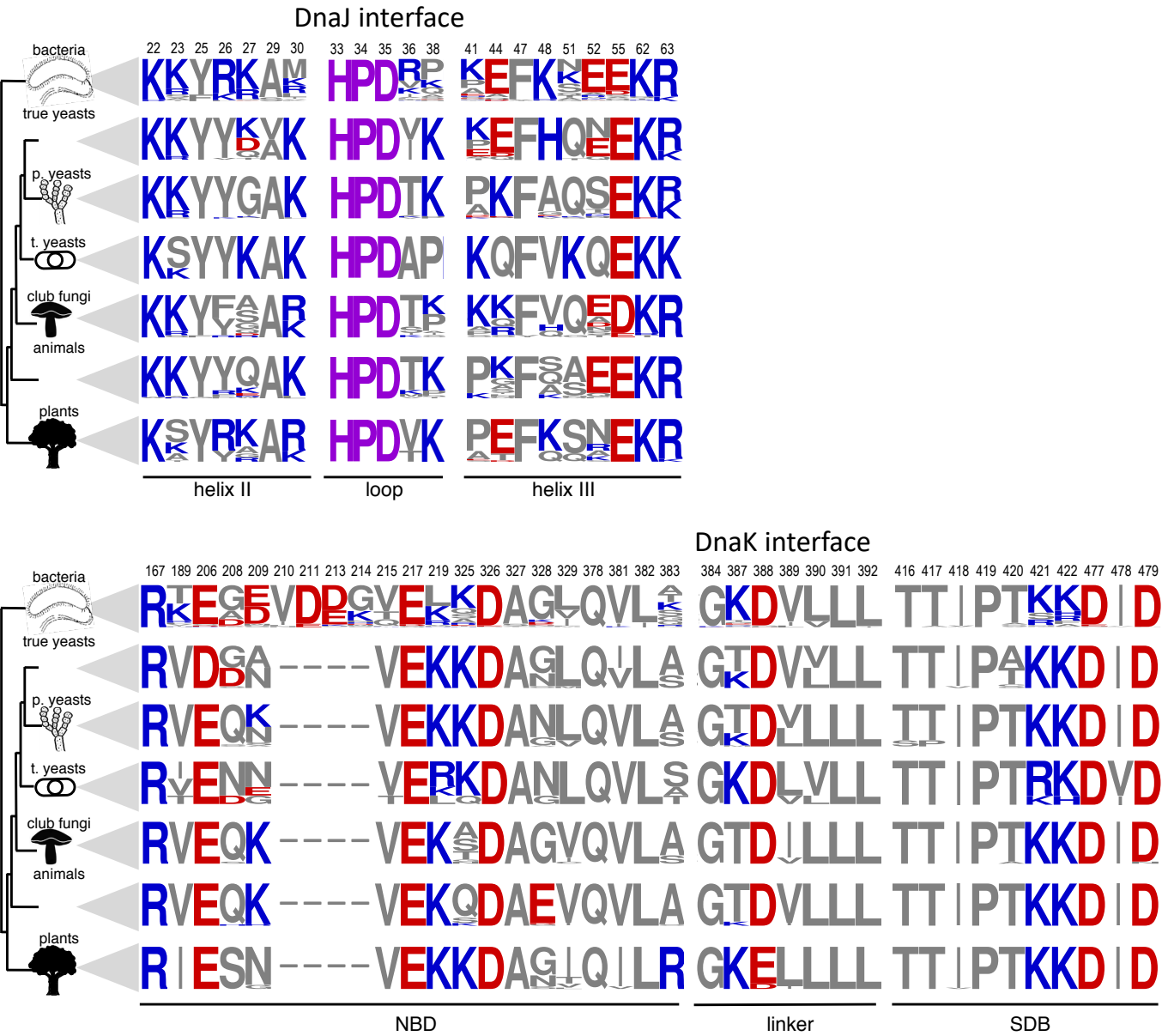

Supplement: S13 Fig — Sequence variability of positions that form the DnaJ/DnaK binding interfaces (positions shown in green in Fig 5A) for orthologs of DnaJ (top) and orthologs of DnaK (bottom) from bacteria and from mitochondria for the following taxonomic groups: Bacteria, Saccharomycotina (true yeasts), Pezizomycotina (p. yeasts), Taphiromycotina (t. yeasts), Basidiomycota (club fungi), Animals (animals) and Plants (plants). Sequence logos represent the amino acid frequency of each interfacial position in the orthologs examined, with positively charged (blue), negatively charged (red) and uncharged (grey) residues. Position numbering is that for E. coli. Phylogenetic relationships among taxonomic groups are depicted on the left. (PDF) [file pcbi.1007913.s013.pdf]

S14 Fig

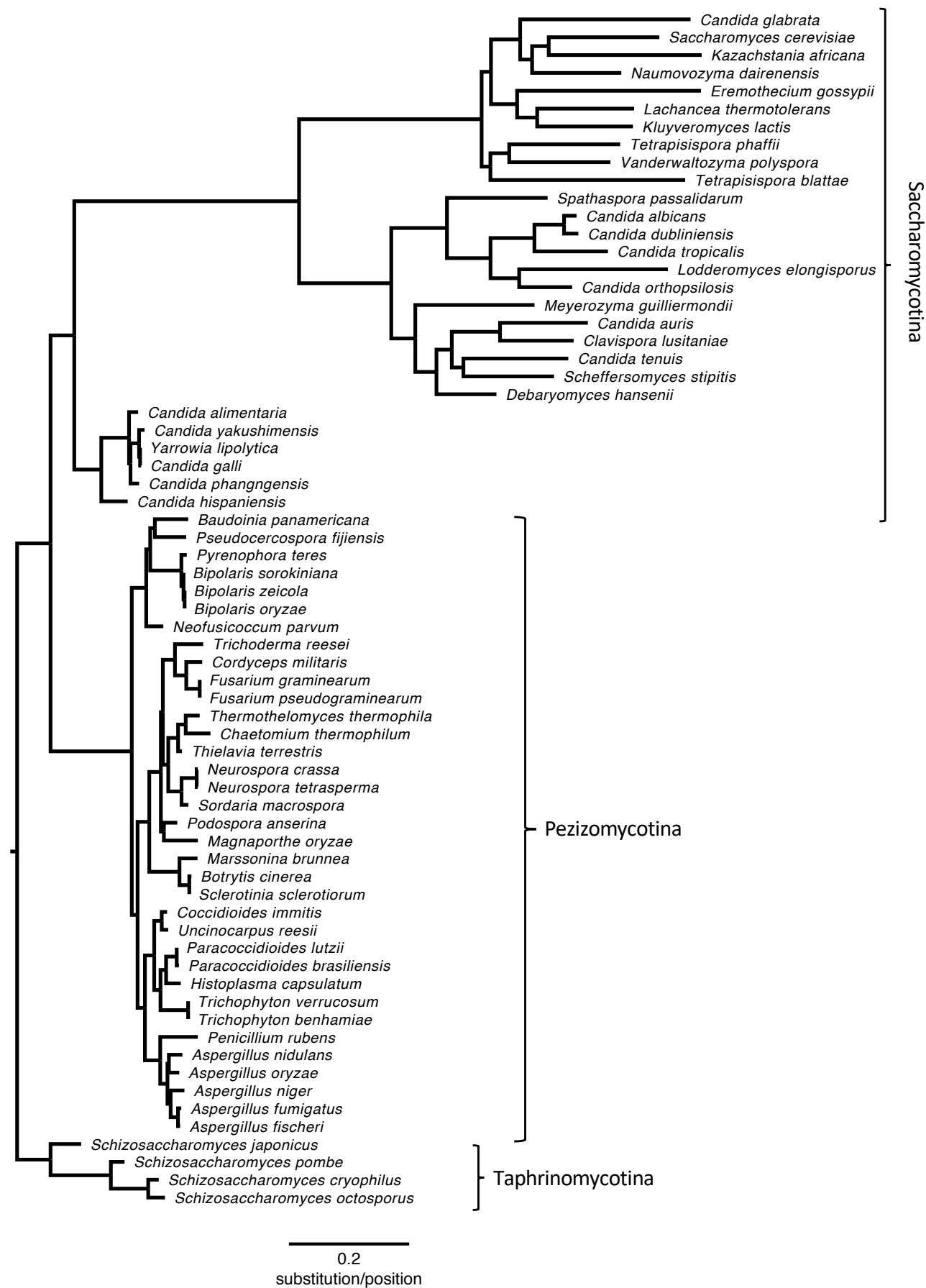

Supplement: S14 Fig — Major clades are indicated. (PDF) [file pcbi.1007913.s014.pdf]

S15 Fig

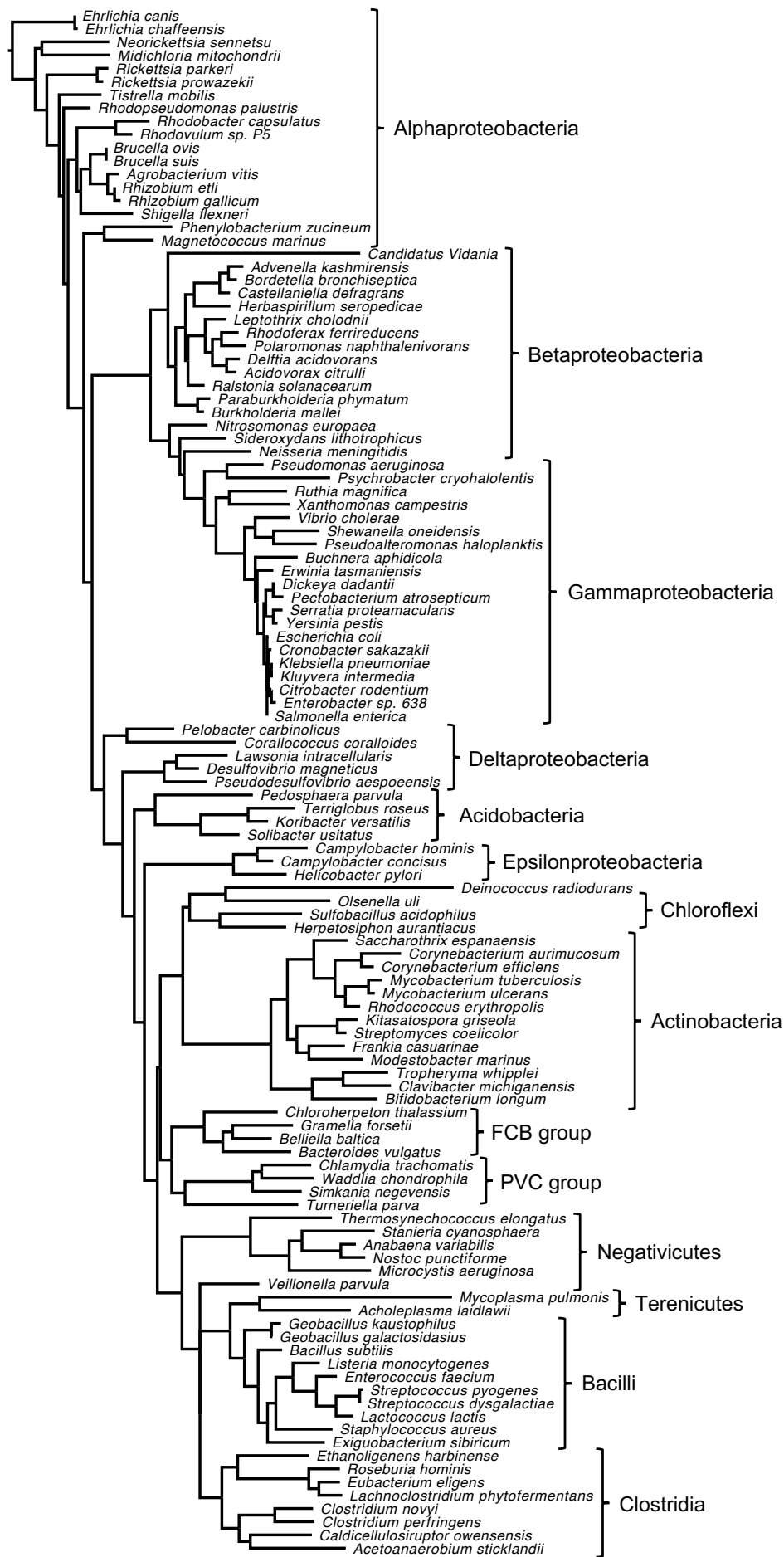

Supplement: S15 Fig — Major clades are indicated. (PDF) [file pcbi.1007913.s015.pdf]

**S16 Fig**

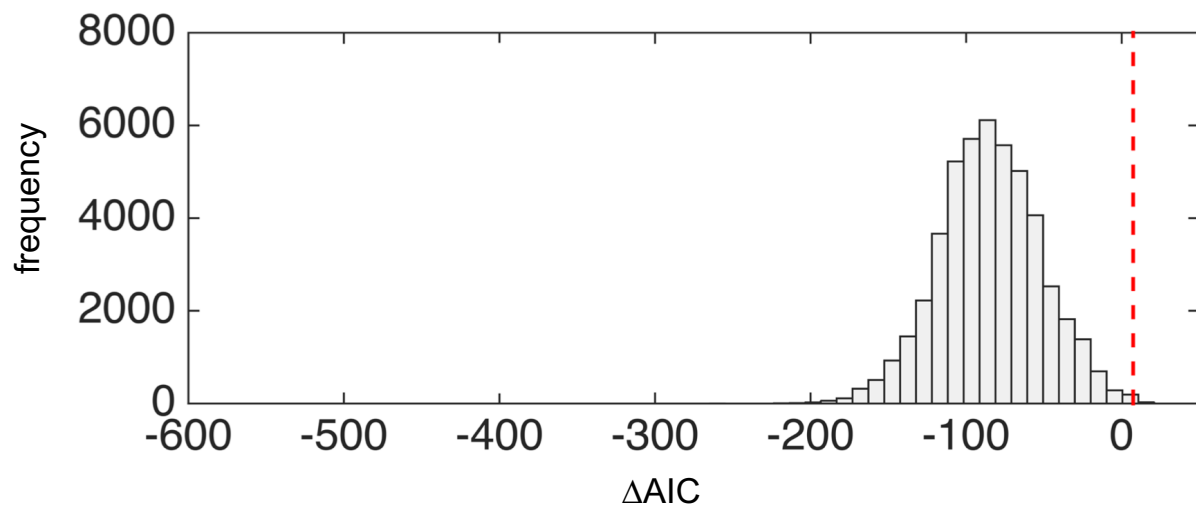

Supplement: S16 Fig — The co-evolution acceptance threshold with p>0.01 confidence was established as ΔAIC = 7.38 (red dashed line). (PDF) [file pcbi.1007913.s016.pdf]

S17 Fig

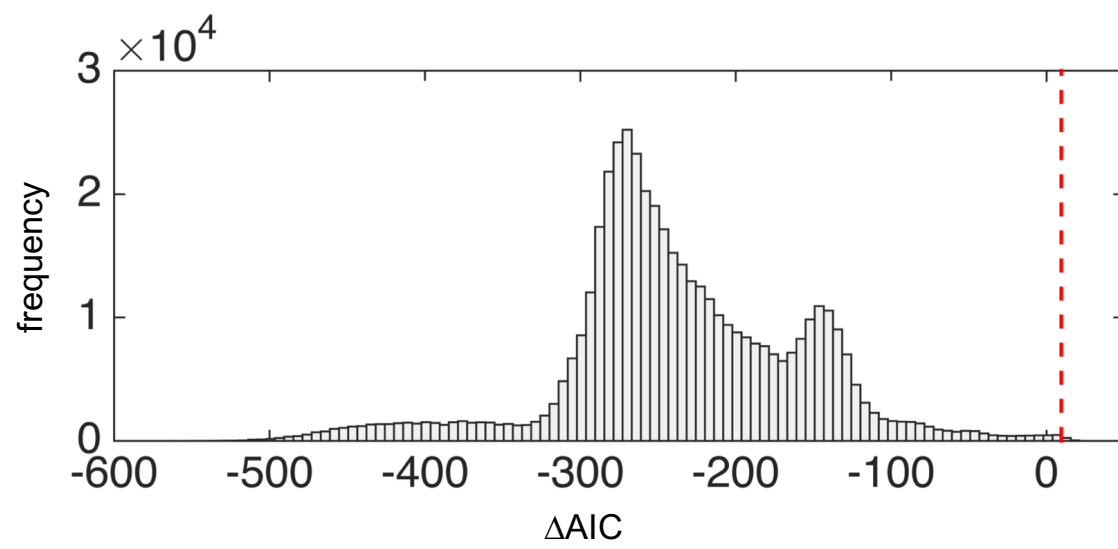

Supplement: S17 Fig — The co-evolution acceptance threshold with p>0.01 confidence was established as ΔAIC = 9.473 (red dashed line). (PDF) [file pcbi.1007913.s017.pdf]
