# Supplementary material for: Two-step mechanism of J-domain action in driving Hsp70 function
Source: PLoS Comput Biol. 2020 Jun 1;16(6):e1007913. doi: 10.1371/journal.pcbi.1007913 (PMC7289447; doi:10.1371/journal.pcbi.1007913)
Supplement: S7 Fig — (a) Probabilities (p) of the most stable ion pairs across the J-domain/Ssq1 interface, calculated from the 10.5 μs trajectory of the dominant bound state. (b) Distance distributions of the ion pairs shown in (a). (PDF) [file pcbi.1007913.s007.pdf]

S7 Fig

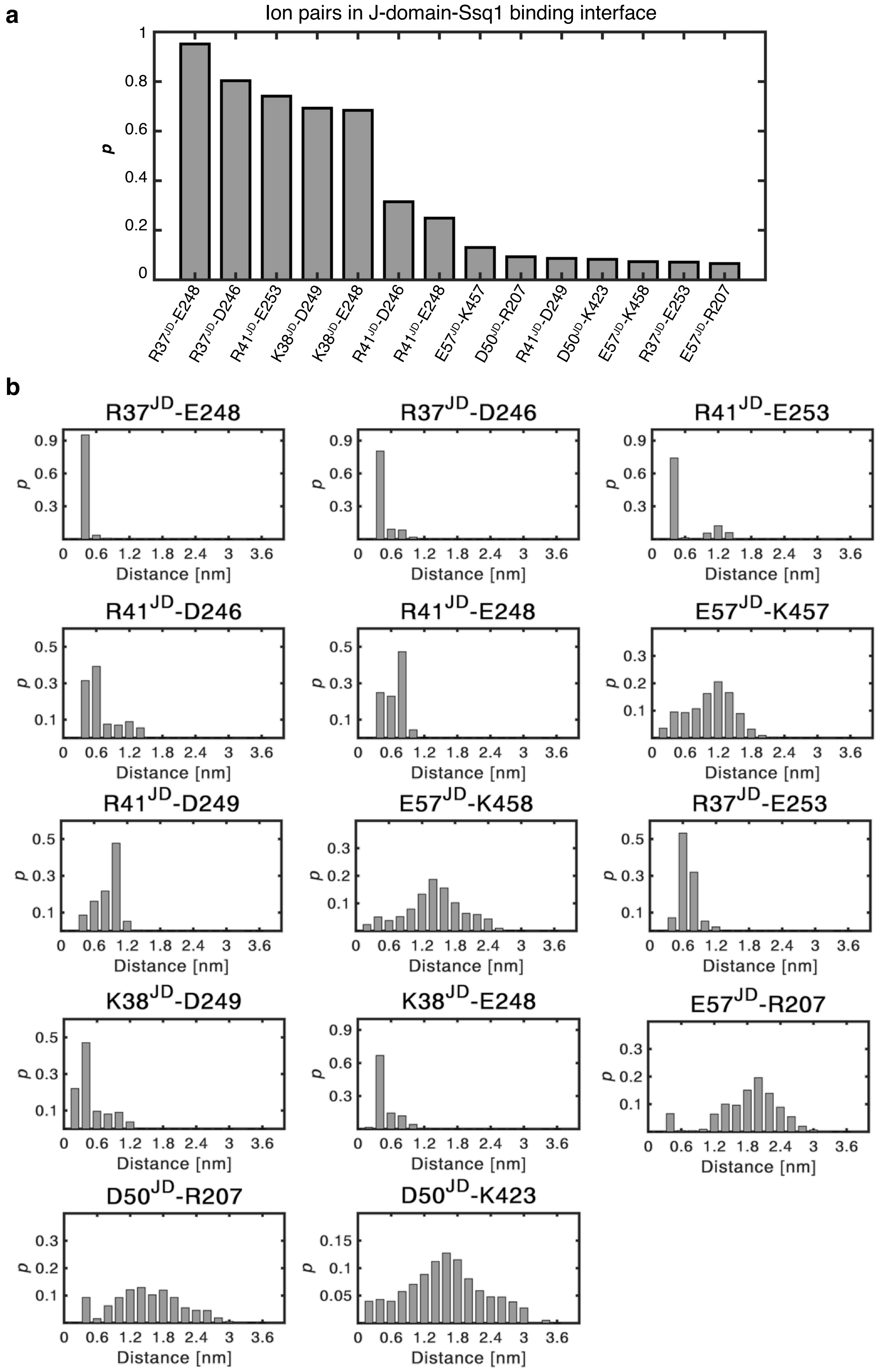
a
Ion pairs in J-domain-Ssq1 binding interface
p
R37JD-E248
R37JD-D246
R41JD-E253
K38JD-D249
K38JD-E248
R41JD-D246
R41JD-E248
E57JD-K457
D50JD-R207
R41JD-D249
D50JD-K423
E57JD-K458
R37JD-E253
E57JD-R207
b
R37JD-E248
R37JD-D246
R41JD-E253
R41JD-D246
R41JD-E248
E57JD-K457
R41JD-D249
E57JD-K458
R37JD-E253
K38JD-D249
K38JD-E248
E57JD-R207
D50JD-R207
D50JD-K423
Distance [nm]
