# Supplementary material for: Two-step mechanism of J-domain action in driving Hsp70 function
Source: PLoS Comput Biol. 2020 Jun 1;16(6):e1007913. doi: 10.1371/journal.pcbi.1007913 (PMC7289447; doi:10.1371/journal.pcbi.1007913)
Supplement: S1 Table — (PDF) [file pcbi.1007913.s018.pdf]

S1 Table | Probability of observing R^{NBD} in four different states

R^{NBD} interacting	Ssq1 alone	Ssq1+J	DnaK alone	Dnak+J
with D^{LK} and D^{SBD}	0.82±0.15	0.62±0.03	0.83±0.05	0.51±0.12
with D^{SBD}	0.08±0.06	0.17±0.05	0.002±0.001	0.14±0.04
with D^{LK}	0.09±0.05	0.20±0.06	0.17±0.06	0.22±0.05
none	0.01±0.007	0.01±0.002	0.0002±0.0001	0.13±0.03

The fraction of each of the four states of R^{NBD} and the standard error of the mean were calculated from 15 independent free energy maps from well-tempered metadymamics as described in Ref. 59
