# Supplementary material for: Two-step mechanism of J-domain action in driving Hsp70 function
Source: PLoS Comput Biol. 2020 Jun 1;16(6):e1007913. doi: 10.1371/journal.pcbi.1007913 (PMC7289447; doi:10.1371/journal.pcbi.1007913)
Supplement: S2 Table — (PDF) [file pcbi.1007913.s019.pdf]

S2 Table | Statistical support for pairs of coevolving Hsc20/Hsp70 positions from Ascomycota.

coevolving positions Hsc20-Hsp70	lnl Coev	AIC* Coev	lnl M0	AIC* M0	ΔAIC	*d/s***
38-248	-118.16	240.33	-134.49	270.97	30.64	8.42
67-249	-157.21	318.42	-172.18	346.36	27.94	3.68
45-249	-162.84	329.69	-174.65	351.30	21.61	10.00
42-456	-171.56	347.12	-182.63	367.27	20.15	5.26
42-248	-166.20	336.41	-176.24	354.48	18.07	5.26
51-249	-173.68	351.36	-183.12	368.23	16.88	4.00
38-249	-165.79	335.58	-175.09	352.18	16.60	5.71
67-456	-128.99	261.98	-137.83	277.66	15.67	10.00
46-248	-169.43	342.87	-177.65	357.31	14.44	5.26
42-457	-164.62	333.25	-172.45	346.90	13.65	10.00
46-456	-176.32	356.64	-184.05	370.10	13.45	5.26
45-248	-126.84	257.69	-133.90	269.81	12.12	10.00
42-424	-164.19	332.37	-170.95	343.90	11.52	5.57
42-453	-161.25	326.50	-167.81	337.62	11.12	10.00
44-248	-67.08	138.17	-73.13	148.26	10.09	8.42
67-248	-125.54	255.09	-131.43	264.87	9.78	17.50

* AIC Akaike information criterion
** The *d/s* ratio represents the strength of coevolution between a pair of position in the Coev model. *s* is the rate at which a coevolving pair is replaced by a non-coevolving pair. *d* is the rate at which the pair of positions returns to the coevolving profile. Therefore, *d/s* represents the attraction of pairs of positions to stay within the coevolving profile. *d/s* = 1 indicates lack of coevolution, *d/s* larger than one indicates the strength of coevolution.
