# Supplementary material for: Two-step mechanism of J-domain action in driving Hsp70 function
Source: PLoS Comput Biol. 2020 Jun 1;16(6):e1007913. doi: 10.1371/journal.pcbi.1007913 (PMC7289447; doi:10.1371/journal.pcbi.1007913)
Supplement: S3 Table — (PDF) [file pcbi.1007913.s020.pdf]

S3 Table | Statistical support for pairs of coevolving DnaJ^{JD}/DnaK positions from bacteria.

coevolving positions DnaJ^{JD}-DnaK	lnl Coev	AIC* Coev	lnl M0	AIC* M0	ΔAIC	*d/s***
51-329	-281.67	567.33	-297.26	596.53	29.19	10.00
38-209	-226.02	456.05	-240.94	483.87	27.83	7.27
26-209	-113.05	230.09	-124.43	250.86	20.77	8.42
23-383	-327.15	658.3	-337.69	677.39	19.09	10.00
38-189	-291.85	587.7	-301.78	605.57	17.87	10.00
23-189	-265.11	534.22	-274.94	551.87	17.65	7.27
36-387	-225.6	455.19	-235.2	472.41	17.21	10.00
36-381	-190.06	384.13	-199.38	400.77	16.64	7.27
62-189	-190.51	385.02	-199.7	401.4	16.38	10.00
38-215	-215.66	435.32	-224.68	451.36	16.04	10.00
38-387	-240.64	485.28	-249.07	500.13	14.85	10.00
51-189	-289.54	583.08	-297.96	597.93	14.85	10.00
52-219	-205.74	415.49	-213.93	429.86	14.37	10.00
55-420	-179.54	363.08	-187.63	377.27	14.18	6.82
23-329	-266.16	536.32	-274.24	550.47	14.15	10.00
51-214	-251.65	507.3	-259.27	520.54	13.25	7.27
36-420	-212.05	428.11	-218.78	439.55	11.45	5.71
52-387	-165.03	334.05	-171.5	345.00	10.95	10.00
52-189	-218.01	440.03	-224.22	450.44	10.41	10.00
62-214	-155.28	314.56	-161.01	324.01	9.45	10.00

* AIC Akaike information criterion
** The *d/s* ratio represents the strength of coevolution between a pair of position in the Coev model. *s* is the rate at which a coevolving pair is replaced by a non-coevolving pair. *d* is the rate at which the pair of positions returns to the coevolving profile. Therefore, *d/s* represents the attraction of pairs of positions to stay within the coevolving profile. *d/s* = 1 indicates lack of coevolution, *d/s* larger than one indicates the strength of coevolution.
