# Supplementary material for: Two-step mechanism of J-domain action in driving Hsp70 function
Source: PLoS Comput Biol. 2020 Jun 1;16(6):e1007913. doi: 10.1371/journal.pcbi.1007913 (PMC7289447; doi:10.1371/journal.pcbi.1007913)
Supplement: S4 Table — (PDF) [file pcbi.1007913.s021.pdf]

S4 Table | Force constants used in Umbrella Sampling for Hsc20-Ssq1 spontaneous binding

Hsc20 WT		Hsc20 R37A R41A	
Distance [nm]	Force constant [kJ/mol * nm^2]	Distance [nm]	Force constant [kJ/mol * nm^2]
1.2	3000	-	-
1.3	3500	1.3	3500
1.4	4000	1.4	4000
1.5	3000	1.5	3000
1.6	3000	1.6	3000
1.7	2500	1.7	2500
1.8	2000	1.8	2000
2.0	1500	2.0	1500
2.2	1000	2.2	1000
2.4	1000	2.4	1000
2.6	1000	2.6	1000
2.8	1000	2.8	1000
3.0	1000	3.0	1000
3.2	1000	3.2	1000
3.4	500	3.4	500
3.6	500	3.6	500
3.8	500	3.8	500
4.0	500	4.0	500
4.2	500	4.2	500
